# Preferences for colorectal cancer screening strategies: a discrete choice experiment

**DOI:** 10.1038/sj.bjc.6605566

**Published:** 2010-03-02

**Authors:** L Hol, E W de Bekker-Grob, L van Dam, B Donkers, E J Kuipers, J D F Habbema, E W Steyerberg, M E van Leerdam, M L Essink-Bot

**Affiliations:** 1 Departments of Gastroenterology and Hepatology, Erasmus MC, University Medical Centre, Rotterdam, The Netherlands; 2 Department of Public Health, Erasmus MC, University Medical Centre, Rotterdam, The Netherlands; 3 Department of Business Economics, Erasmus School of Economics, Erasmus University, Rotterdam, The Netherlands; 4 Department of Internal Medicine, Erasmus MC, University Medical Centre, Rotterdam, The Netherlands; 5 Department of Social Medicine, Academic Medical Centre, Amsterdam, The Netherlands

**Keywords:** colorectal cancer, screening, preference, discrete choice experiment

## Abstract

**Background:**

Guidelines underline the role of individual preferences in the selection of a screening test, as insufficient evidence is available to recommend one screening test over another. We conducted a study to determine the preferences of individuals and to predict uptake for colorectal cancer (CRC) screening programmes using various screening tests.

**Methods:**

A discrete choice experiment (DCE) questionnaire was distributed among naive subjects, yet to be screened, and previously screened subjects, aged 50–75 years. Subjects were asked to choose between scenarios on the basis of faecal occult blood test (FOBT), flexible sigmoidoscopy (FS), total colonoscopy (TC) with various test-specific screening intervals and mortality reductions, and no screening (opt-out).

**Results:**

In total, 489 out of 1498 (33%) screening-naïve subjects (52% male; mean age±s.d. 61±7 years) and 545 out of 769 (71%) previously screened subjects (52% male; mean age±s.d. 61±6 years) returned the questionnaire. The type of screening test, screening interval, and risk reduction of CRC-related mortality influenced subjects’ preferences (all *P*<0.05). Screening-naive and previously screened subjects equally preferred 5-yearly FS and 10-yearly TC (*P*=0.24; *P*=0.11), but favoured both strategies to annual FOBT screening (all *P*-values <0.001) if, based on the literature, realistic risk reduction of CRC-related mortality was applied. Screening-naive and previously screened subjects were willing to undergo a 10-yearly TC instead of a 5-yearly FS to obtain an additional risk reduction of CRC-related mortality of 45% (*P*<0.001).

**Conclusion:**

These data provide insight into the extent by which interval and risk reduction of CRC-related mortality affect preferences for CRC screening tests. Assuming realistic test characteristics, subjects in the target population preferred endoscopic screening over FOBT screening, partly, due to the more favourable risk reduction of CRC-related mortality by endoscopy screening. Increasing the knowledge of potential screenees regarding risk reduction by different screening strategies is, therefore, warranted to prevent unrealistic expectations and to optimise informed choice.

Colorectal cancer (CRC) is the second leading cause of cancer-related deaths in the Western world. Screening can reduce CRC-related mortality by removal of adenomas and early detection of CRC (Newcomb *et al*, 1992; Selby *et al*, 1992; Mandel *et al*, 1993; Hardcastle *et al*, 1996; Kronborg *et al*, 1996). There is compelling evidence to support screening of average-risk individuals aged over 50 years (Mandel *et al*, 1993; Kewenter *et al*, 1994; Hardcastle *et al*, 1996; Kronborg *et al*, 1996; Hoff *et al*, 2009). Guidelines underline the role of individual preferences in the selection of a screening test (Council Recommendation on Cancer Screening, 2003; Levin *et al*, 2008; Sung *et al*, 2008), as insufficient evidence is available to recommend one screening test over another. Individual preferences for a certain screening test have been found to influence uptake in a CRC-screening programme (Wolf *et al*, 2006). Uptake is a key factor that determines the effectiveness of such a screening program. However, uptake levels are fairly low in many countries (<60%) (Hardcastle *et al*, 1996; Kronborg *et al*, 1996; Segnan *et al*, 2002; UK Flexible Sigmoidoscopy Screening Investigators, 2002; van Rossum *et al*, 2008; Hol *et al*, 2009a). Several countries, including The Netherlands, are presently considering to introduce a nation-wide CRC-screening program. It is therefore essential to obtain insight into individual preferences for available screening strategies before the implementation of a nation-wide screening programme.

Previous surveys have demonstrated a broad variation in preferences for CRC screening tests, as tests differ in benefit (CRC mortality reduction) on the one hand and potential harms on the other hand (perceived burden and complications). Subjects who valued effectiveness most highly chose colonoscopy screening, whereas others preferred faecal occult blood testing (FOBT) because of its less invasive nature (Pignone *et al*, 1999; Frew *et al*, 2005; Wolf *et al*, 2006; DeBourcy *et al*, 2008). These studies, however, did not provide data on the relative importance of test characteristics on preferences; for example, how much potential health gain does a subject require to undergo invasive endoscopic screening?

Discrete choice experiments (DCEs) are becoming more widely used in health-care research (Gyrd-Hansen and Sogaard, 2001; Sculpher *et al*, 2004; Marshall *et al*, 2007, 2009; Hur *et al*, 2008). A DCE is capable of establishing preferences and to predict uptake in controlled experimental conditions, through responses to realistic and hypothetical scenarios. The DCEs may be valuable for patient-centred evaluations of health technologies (Ryan, 2004).

This study was conducted to determine individuals’ preferences and to predict the uptake of CRC screening programmes with various screening tests, and the relative importance of different test characteristics for these preferences in an average-risk population. Furthermore, we aimed to identify the differences in preference structures among subgroups in the population.

## Materials and methods

### Study population

**Figure 1:**
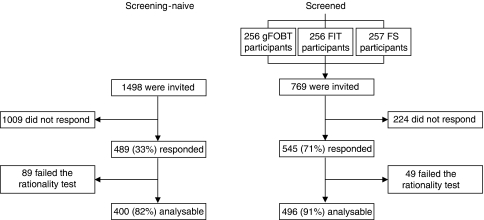
Study profile. gFOBT=guaiac-based faecal occult blood test; FIT=immunochemical faecal occult blood test; FS=flexible sigmoidoscopy.

A total of 1498 screening-naive individuals, aged 50–74 years, were randomly selected from municipal registries of the Rotterdam region in the southwest of the Netherlands. We also invited a random sample of 769 screened subjects of a CRC screening trial comparing guaiac-based FOBT (gFOBT), faecal immunochemical test (FIT) and flexible sigmoidoscopy (FS; Figure 1). This screening trial was carried out in the same target population as mentioned above (Hol *et al*, 2009a). Age, sex and social economic status were found to be equally distributed among the screening-naive and previously screened invitees.

### Discrete choice experiments

The DCEs can measure individuals’ preferences for health-care interventions. The DCEs are based on the assumptions that a health-care intervention can be described by its characteristics (attributes) (e.g. frequency of undergoing the intervention) and that the individual valuation of the intervention is determined by pre-defined levels (e.g. monthly or yearly) of those attributes. The health-care intervention (e.g. screening test) and its test characteristics have to be specified before generating an experimental design. In a DCE, individuals choose between several realistic and hypothetical scenarios. Preference estimates can be obtained from the choice data and describe the relative preference for characteristics of the health-care intervention.

### Attributes and levels

Tests such as FOBT, FS and total colonoscopy (TC) are most widely used as CRC screening tests and, therefore, are incorporated in this study design. The characteristics and their levels were derived from the literature, expert opinions (*n*=3) and interviews with potential screenees (*n*=40). Experts were asked to comment on a list of characteristics derived from literature review. Potential screenees could also comment on the list of characteristics and rank them in the order of importance. On the basis of these data, we selected the two most important characteristics as identified by both groups: risk reduction of CRC-related mortality (RR) and screening interval. Notably, characteristics that are related directly to the test (e.g. oral bowel cleansing solution is not required for FOBT and always for TC) were already captured by the specific screening test (FOBT, FS and TC). All subjects were informed regarding the incorporated test characteristics of the three screening tests (Table A2). The specific values (levels; e.g. amount of risk reduction or length of screening interval) for each test characteristic incorporated the range of possible test outcomes of a specific screening test (FOBT, FS and TC) based on the current literature (Table 1). The levels were test-specific to create realistic scenarios (Table 1). Levels of RR were presented in the questionnaire as absolute values to reduce framing effects, in accordance to the literature (Edwards *et al*, 2002). In the presentation of the results in this paper, we used the relative risk on CRC-related death, as this is most commonly used in the screening literature (e.g. FOBT: 13–18% RR). The absolute risk of CRC-related death without screening was set at 3.0%. People aged 50 years in the Netherlands have a 3.0% risk of dying from CRC, based on data from the Dutch comprehensive cancer centre (IKC, www.ikc.nl).

### Study design and questionnaire

The design contained three tests (FOBT, FS and TC) and two characteristics (RR of CRC-related mortality and screening interval) with three levels each (Table 1). The test-specific levels (e.g. screening interval of FOBT between 4 months and triennial) were required to select realistic combinations. Furthermore, unrealistic combinations of the characteristics’ levels were blocked (i.e. a combination of the lowest RR with the shortest screening interval and the highest RR combined with the longest screening interval). The combination of the characteristics and levels resulted in 21 (i.e. 7 × 3) possible test scenarios, and thus 343 (i.e. 7^3^) possible combinations of scenarios (i.e. full factorial design). It is not feasible to present a single individual with all these combinations. We therefore reduced the design in such away that two-way interactions could be estimated (i.e. we created a fractional factorial design). We therefore used SAS software (Version 9.1, SAS Institute, Cary, NC, USA) that is capable of generating designs that are highly efficient (i.e. maximising D-efficiency or minimising D-error) in such circumstances (Street *et al*, 2005). We chose a design with 84 choice sets divided over seven versions of the questionnaire (D-error: 0.573). Each choice set included two CRC screening tests and an option of not to be screened (opt-out; Table A1). A design in which all three screening tests and the option not to be screened were presented in one scenario was not feasible, as the pilot study (*n*=20) showed a significant decrease in subjects’ understanding and acceptance of the questionnaire.

A rationality test was included in the questionnaire to determine the understanding of the questionnaire by each subject. The rationality test was a choice set of which one screening option was logically preferred over the other option given the levels of each test characteristic (biennial FS screening resulting in 40% RR against biennial FS screening resulting in 70% RR). It is common practice to exclude irrational responses (Weston and Fitzgerald, 2004; Ryan *et al*, 2005; Langenhoff *et al*, 2007), and we therefore adopted this approach. However, some recent discussions in the literature suggest that these responses could be included (Lancsar and Louviere, 2006; Ryan *et al*, 2009). Furthermore, sensitivity analyses were conducted and inclusion of irrational responses led to similar results.

Subjects’ social economic status (SES), previous lower endoscopy experience (sigmoidoscopy or colonoscopy) and experience with CRC in family or close friends were determined. Furthermore, the generic health status (EQ-5D summary score) was assessed. This is a validated classification of subject's own health (Dolan, 1997).

We conducted a pilot study (*n*=20) to ascertain that subjects could manage the length of the questionnaire and to evaluate subjects’ understanding, acceptance and face validity of the questionnaire, and the background information on the three screening tests (Table A2). The questionnaire was mailed to all subjects. Background information on the three screening tests was printed on the first page of the questionnaire (Table A2). A reminder was sent to non-responders 4 weeks later.

### Data analysis

Each choice between two tests and the opt-out was considered as a specific observation. The DCE was analysed using multinomial logit regression models with test-specific parameters. The model was implemented in SAS software (Version 9.1, SAS Institute). *A priori* we expected the test and the two characteristics to be important for subjects’ choices and that a higher RR value and lengthening of ‘screening interval’ would have a positive effect on preferences.

We assumed that there was no linear relationship between the different levels of the characteristics. Therefore, we estimated the following models for the DCE:



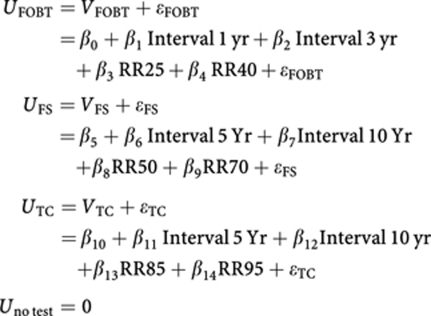



Utility (*U*) represents the preference for a (hypothetical) CRC screening programme. Utility consists of the deterministic and observable component (*V*) and the random component (*ε*) of the analysis, accounting for unobserved or unobservable components of choice. The observed utility (*V*) in this study is referred to as preference (*V*). The absolute value of *V* has a relative interpretation: the higher the value of *V*, the stronger a respondent's preference for a particular screening strategy. The constant terms (screening test; *β*_0_, *β*_5_ and *β*_10_) are alternative specific constants that indicate the general attitude of subjects towards screening with a specific screening test compared with no screening. *β*_1,2_, *β*_6,7_, and *β*_11,12_ are coefficients of the levels of the test characteristic ‘screening interval’ and *β*_3,4_, *β*_8,9_, and *β*_13,14_ are coefficients of the levels of the test characteristic ‘risk reduction of CRC related mortality’ each coefficient indicates the relative weight individuals place on that test specific level compared with the reference level for that test-specific test characteristic (for reference levels see Table 3). A two-sided *P*-value smaller than 0.05 was considered to be statistically significant.

Generic health status was dichotomised to an EQ-5D summary score of ‘1’, representing full health, versus an EQ-5D summary score ‘<1’, indicating sub-optimal health. Aggregate data on SES were available at the level of the area postal code (www.cbs.nl) of the subject, weighted by population size and classified into three groups (high, intermediate and low).

Chi-square and Student's *t*-tests were used to assess the differences in the value of characteristics between screening-naive and screened subjects, as well as among subgroups (age, gender, SES, EQ-5D, prior endoscopy experience or knowing someone affected by CRC) within the screening-naive population.

To examine the predicted uptake of CRC screening based on our results, we applied previously proposed models to our data (Hall *et al*, 2002; Gerard *et al*, 2008). We also investigated the effect of changing the characteristics, as identified by the results of our multinomial logit model, on the expected uptake of CRC screening.

### Ethical approval

The study was approved by the Institutional Review Board of the Erasmus MC, University Medical Centre (MEC-2007–224).

## Results

A total of 489 out of 1498 (33%) screening-naive and 545 out of 769 (71%) previously screened subjects returned the questionnaire. Screening-naive subjects were of higher SES than screened subjects (*P*<0.001, Table 2). A higher proportion of screened subjects previously underwent a lower endoscopy compared with screening-naive subjects (49 *vs* 23% *P*<0.001). Among the subjects that participated in the CRC screening trial, all (172 out of 172) FS screenees and 22% (70 out of 324) of the screenees (those had undergone a FOBT previously) underwent a lower endoscopy.

### DCE

A significantly higher proportion of the previously screened subjects (91%) passed the rationality test compared with the screening-naive subjects (82% *P*<0.001).

**Figure 2:**
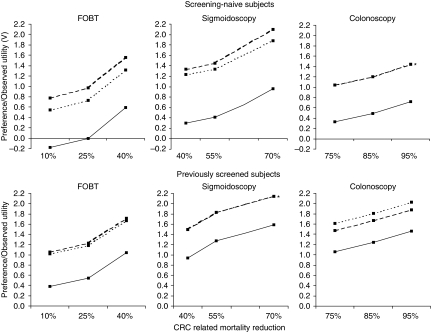
Preferences for the different screening strategies at a long (–––), intermediate (---) and short (—) screening interval and different levels of mortality risk reduction for screening-naive and previously screened subjects. FOBT=faecal occult blood test; CRC=colorectal cancer. ^*^Preferences for long and intermediate screening interval were similar.

Screening-naive subjects did not prefer FOBT over no screening. They expressed a positive attitude towards FS and TC (positive and statistically significant sign; Table 3; Figure 2). A high RR was preferred over intermediate and low RR for all screening tests (*P*-values <0.01). Screening-naive subjects expressed a more positive attitude towards an intermediate (FOBT: annually; FS: 5 yearly; TC: 5 yearly) compared with a short screening interval (FOBT: 4 monthly; FS: annual; TC: biennial). Further lengthening of the screening interval (FOBT: triennial; FS: 10 yearly; TC: 10 yearly) had only a small positive effect on subjects’ preferences for FOBT (*P*=0.02) and FS (*P*=0.02), and no effect on subjects’ preferences for TC screening.

Previously screened subjects had a positive attitude towards all screening tests (*P*<0.001). A high RR value was preferred over intermediate and low RR for all screening tests, and an intermediate screening interval was preferred over a short screening interval (Table 3, Figure 2). Previously screened subjects did not prefer an intermediate interval over a long interval for all screening tests (FOBT: *P*=0.67; FS: *P*=0.99; TC: *P*=0.10).

### Screening-naive versus previously screened subjects

Previously screened subjects had a more positive attitude towards all screening tests than screening-naive subjects (Table 3, *P*<0.001). The differences in preferences regarding RR and screening interval between screening-naive and screened subjects were statistically not significant, except for preferences regarding 5- and 10-yearly FS screening. The more positive attitude of screening-naive subjects towards longer screening intervals (5 yearly *P*<0.001; 10 yearly *P*<0.001) indicated that screening-naive subjects valued infrequent screening more positively than screened subjects.

### Differences in preferences between subgroups

No differences in preferences were found between men and women, apart from a more positive attitude towards TC among men (TC: *P*=0.02). Men, in contrast to women, did prefer FS and TC to no screening (men: FS: *P*<0.001; TC: *P*<0.001; women FS: *P*=0.07; TC: *P*=0.84). Respondents’ age, SES and EQ-5D summary score did not influence the attitude towards a screening test, interval or RR. Subjects who reported to have a close friend or family member with CRC expressed a more positive attitude towards TC screening than subjects without (*P*=0.01). Experience with FS or TC was positively associated with the willingness to undergo a TC (*P*<0.001). Subjects that underwent FS screening had a more positive attitude towards FS and TC screening than subjects who performed a FOBT (*P*<0.001).

### Trade-offs

Screening-naive subjects were, when assuming the same interval (annual) and RR (40%), more willing to undergo FOBT than FS screening (preference/observed utility (*V*) FOBT: *V*=1.32; FS: *V*=0.30; *P*<0.001). Preferences were similar for a 5-yearly FS and an annual FOBT if both tests would generate a RR of 40% (FOBT: *V*=1.32; FS: *V*=1.23; *P*=0.40). A 5-yearly FS was preferred to annual FOBT if FOBT was associated with a less favourable RR than FS screening (FOBT 25% RR: *V*=0.73; FS 40% RR: *V*=1.23; *P*<0.001).

A 5-yearly FS was preferred to a 10-yearly TC if the difference in RR was 25% in favour of TC (e.g. FS: RR 50% TC: RR 75% *P*<0.001). The preferences for a 5-yearly FS and a 10-yearly TC were similar if TC would achieve an additional 35% RR (*P*=0.24), whereas more than 45% difference in RR was associated with a preference for 10-yearly TC (e.g. FS: RR 50% TC: RR 95% *P*<0.001).

Screening-naive subjects equally preferred FS and TC screening, but did prefer both endoscopic screening options over FOBT screening if, based on the literature, the most realistic screening intervals and mortality reduction were applied (annual FOBT RR 25%: *V*=0.73; 5-yearly FS RR 50%: *V*=1.33; 10-yearly colonoscopy RR 85%: *V*=1.22 ; FS *vs* FOBT *P*<0.001; TC *vs* FOBT *P*<0.001; TC *vs* FS *P*=0.24).

Screened subjects made similar trade-offs between the screening test, interval and RR as screening-naive subjects.

### Predicted uptake

**Figure 3:**
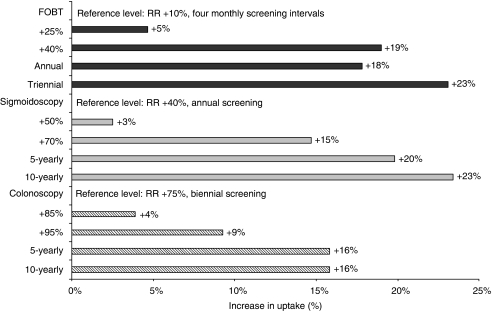
Effects of changing the screening programme characteristics on the average probability of uptake for, respectively, FOBT (45%), FS (58%) and TC (58%) in screening-naive subjects.

Predicted uptake of screening-naive subjects for FOBT, FS and TC screening was 45, 58 and 58, respectively, assuming screening with the reference level for RR and screening interval. On the basis of realistic screening intervals and mortality reduction from the literature, these numbers were 68% for FOBT, 79% for FS and 77% for TC. The screening programme characteristics had substantial impact on the expected uptake among screening-naive subjects (Figure 3).

## Discussion

### Principle findings

In this population-based study, we observed that the type of screening test, screening interval and RR of CRC-related mortality significantly influenced individual preferences among screening-naive and previously screened subjects in the target population (aged 50–74 years). These data provide insight into the relative importance the effect of screening interval and RR of CRC-related mortality on preferences for the three most commonly used screening tests. Both screened and screening-naive subjects preferred FS and TC to FOBT screening if, based on the literature the most realistic screening interval and RR on CRC-related mortality were applied (annual FOBT with 25% RR; 5-yearly FS with 50% RR; 10-yearly colonoscopy with 85% RR; Mandel *et al*, 1993; Winawer *et al*, 1993; Kewenter *et al*, 1994; Hardcastle *et al*, 1996; Kronborg *et al*, 1996; Faivre *et al*, 2004; Levin *et al*, 2008; Hoff *et al*, 2009). This underlines the importance of adequate information on those aspects of CRC screening to achieve informed decision making by potential screenees.

Five studies investigated preferences in CRC screening using a DCE (Gyrd-Hansen and Sogaard, 2001; Salkeld *et al*, 2003; Marshall *et al*, 2007; Hawley *et al*, 2008; Howard and Salkeld, 2008), with two studies investigating preferences among available screening tests (Marshall *et al*, 2007; Hawley *et al*, 2008). This is the first DCE including both a screening-naive and previously screened population. In agreement with previous DCE studies, we found that RR dominated preferences for a screening test. Both FS and TC screening were therefore preferred over FOBT screening when associated with sufficient RR (Marshall *et al*, 2007; Hawley *et al*, 2008).

The literature on preferences for the optimal screening interval per test is limited. One study reported a preference for 5 or 10 yearly to annual screening irrespective of the screening test (Hawley *et al*, 2008). However, deciding on screening interval without information on the screening test leads to unrealistic choices, as an annual FOBT is less burdensome than an annual TC. We therefore used test-specific screening intervals that add to the validity of our results. In our study, previously screened subjects equally preferred intermediate and long screening interval for all tests. Reassurance may be a reason for preferring frequent screening (Cantor *et al*, 2002). However, both intermediate and long interval of all three screening tests were preferred over a short interval, suggesting that subjects trade-off between reassurance and frequency of undergoing a screening test.

Men had a more positive attitude towards FS and colonoscopy screening than women. This finding is in accordance with FS screening programmes that described a lower uptake among women than among men (Segnan *et al*, 2002; UK Flexible Sigmoidoscopy Screening Investigators, 2002; Hol *et al*, 2009a). Known barriers for women to participate in endoscopy screening are male endoscopists (Menees *et al*, 2005) and anxiety before screening (Farraye *et al*, 2004). A different approach to inform both sexes on screening or sex-specific screening strategies might be considered in a nation-wide screening programme to improve acceptance.

The results of this study may be relevant to predict population preferences for newer screening tests with a similar profile or an improved version of a screening test. For example, recently randomised trials demonstrated more favourable detection rates for FIT than gFOBT (van Rossum *et al*, 2008; Hol *et al*, 2009b), suggesting a larger reduction of CRC-related mortality. According to our data, informing people in the target population about a more favourable effect on CRC-related mortality of FIT would lead to a higher acceptance of FIT screening and most likely a higher uptake.

Predicted uptake of FS or TC screening based on our model was significantly higher than uptake of FOBT screening, given realistic levels. This finding is in contrast to the observed higher uptake of FOBT than FS screening in the randomised screening trial performed in the same population as this DCE. Screenees in this trial were, however, not specifically informed on test efficacy. This suggests that increasing awareness on the efficacy of a screening test may enhance uptake. It is therefore of paramount importance to improve the level of awareness on achievable risk reduction of CRC-related mortality to obtain a higher uptake, especially for the more effective endoscopic screening tests. This is further underlined by two European studies. A Swiss study (Marbet *et al*, 2008), in which the majority (75%) of all screenees chose to undergo a TC, and only a small proportion (25%) preferred FOBT or FS screening after they were informed about the efficacy of the three screening tests. A large population-based Italian study found similar participation rates for FS and FOBT when subjects were offered a choice between both strategies (Segnan *et al*, 2005).

### Strengths and weaknesses of this study

In contrast to previous DCE studies, we used a labelled instead of an unlabelled DCE design. In a labelled design, the specific screening test is mentioned in each choice option (FOBT, FS and TC; Table A1), whereas in an unlabelled design the screening test is presented as ‘screening test ‘A’, ‘B’ or ‘C’ and is further described by certain characteristics that are presented in the choice set. CRC screening tests may evoke individual feelings that cannot be described in a questionnaire (e.g. anxiety for an endoscopy). It is therefore difficult to adequately convey the essential differences from a subject's perspective between FOBT and endoscopic tests in terms of, for example, ‘more burdensome’ or ‘less burdensome’. Using a labelled design, scenarios are more realistic, which adds to the validity of the results. Furthermore, we assessed preferences among screening-naive and screened subjects within the target population (aged 50–74 years), including all social economic classes that add to the generalisability of the results. Experienced subjects stated a more positive attitude towards all screening tests than screening-naive subjects. A selection bias may explain this difference in attitude, as experienced subjects have already demonstrated interest in screening and therefore express a more positive attitude towards screening. There is, however, also an experience effect, that is, anticipated discomfort and pain might be higher than actually experienced. This experience might reduce anticipated pain and discomfort for successive screening round. In addition, there may also be an expose effect, that is, people tend to develop a preference merely because they are familiar with it. Our results suggest that subjects who underwent screening are willing to return for a successive screening round that is of vital importance for the efficacy of a screening program. Costs of screening were not included as a test characteristic in this study. All CRC screening programs in Europe, including the Netherlands, do not require out-of-pocket costs. Including cost would therefore influence the results in an unrealistic manner. A limitation of this study is the significantly lower response rate in screening-naive than in previously screened subjects. This may have led to selection bias. Non-respondents may have a more negative attitude towards screening than respondents. Our results may therefore reflect a more positive attitude than that exists in the general population as a whole. The method of framing the levels of risk reduction may have influenced our results. However, we minimised the framing effect in accordance to the literature by presenting absolute values in the questionnaire (Edwards *et al*, 2002). It is common practice to exclude irrational responses from the analysis (Weston and Fitzgerald, 2004; Ryan *et al*, 2005; Langenhoff *et al*, 2007), and that was why this approach was adopted here. Ryan *et al* (2009) recently postulated that researchers should be cautious when excluding respondents, who failed the rationality test. Additional information on respondents’ considerations for failing the rationality test is required. The usage of a ‘think aloud technique’ in the group of subjects who failed the rationality test to determine truly irrational responses has been suggested (Lancsar and Louviere, 2006; Ryan *et al*, 2009). Further research on the effects of excluding subjects based on additional information on failing the rationality test is needed to adopt this approach as common practice.

## Conclusions

These data provide insight into the extent by which interval and RR of CRC-related mortality affect preferences for CRC screening tests in the experienced and screening-naive subjects. Both screening-naive and previously screened subjects stated a more positive attitude towards both endoscopic screening strategies than FOBT if, based on the literature, the most realistic screening interval and RR on CRC-related mortality were applied. The RR value of CRC-related mortality determined preferences for endoscopic screening. This underlines the importance of awareness on achievable RR of CRC-related mortality of the different screening test to enhance uptake particularly for endoscopic screening tests and to optimise informed choice.

## Figures and Tables

**Table 1:** Alternatives, attributes and the alternative specific levels based on the literature

**Alternatives**	**Alternative specific levels**	**Literature**	**References**
*Screening interval (year)*			
FOBT	1/3–1–3	1–2	(Frazier *et al*, 2000; Levin *et al*, 2008)
Sigmoidoscopy	1–5–10	5–10	(Levin *et al*, 2008; Frazier *et al*, 2000)
Colonoscopy	2–5–10	5–10	(Frazier *et al*, 2000; Levin *et al*, 2008)
			
*Risk reduction* (%)			
FOBT	10–25–40	13–33	(Kronborg *et al*, 1996; Hardcastle *et al*, 1996; Kewenter *et al*, 1994; Mandel *et al*, 1993)
Sigmoidoscopy	40–50–70	49–62	(Selby *et al*, 1992; Zauber *et al*, 2008; Newcomb *et al*, 1992; Muller and Sonnenberg, 1995; Hoff *et al*, 2009)
Colonoscopy	75–85–95	80–84	(Winawer *et al*, 1993; Zauber *et al*, 2008)

Abbreviation: FOBT=faecal occult blood test.

**Table 2:** Subjects’ characteristics

	**Screening- naive subjects**	**Previously screened subjects**	***P*-value**
Analysable subjects	400	496	
Sex (male; *n* (%))	209 (52)	260 (52)	0.96
Age (mean (s.d.))	60.7 (6.6)	61.1 (6.4)	0.36
EQ5D score (mean (s.d.))	0.94 (0.11)	0.93 (0.10)	0.76
*Social economic status* (*n* (%))			<0.01
High	195 (49)	196 (40)	
Intermediate	77 (19)	96 (19)	
Low	128 (32)	204 (41)	
*Lower endoscopy experience (n (%))*			<0.01
Yes	92 (23)	242 (49)	
No	307 (76)	251 (50)	
Unknown	1 (1)	3 (1)	
*Knowing someone affected by CRC (n (%))*			0.78
Yes	53 (13)	67 (13)	
No	285 (71)	381 (77)	
Unknown	62 (16)	48 (10)	

**Table 3:** Regression coefficients from the discrete choice experiments for the different tests and attributes

**Attribute levels**	**Screening-naive subjects Coefficient**	**(95%CI)**	**Previously screened subjects Coefficient**	**(95%CI)**	***P*-value** ^†^
*Screening test (base level ‘no screening’)*					
No screening *(reference level)*					
FOBT	−0.18	(−0.44;0.08)	0.38	(0.15;0.62)^*^	<0.001
Sigmoidoscopy	0.30	(0.06;0.54)^*^	0.94	(0.72;1.16)^*^	<0.001
Colonoscopy	0.33	(0.08;0.57)^*^	1.05	(0.84;1.27)^*^	<0.001
					
*Risk reduction of CRC-related mortality*					
*FOBT*					
*3–2.7% (RR 10%) (reference level)*					
3–2.4% (RR 25%)	0.19	(−0.01;0.38)	0.17	(-0.01;0.34)	0.88
3–1.8% (RR 40%)	0.78	(0.54;1.02)^*^	0.65	(0.44;0.87)^*^	0.45
					
*Sigmoidoscopy*					
*3.0–1.8% (RR 40%) (reference level)*					
3.0–1.5% (RR 50%)	0.10	(−0.09;0.29)	0.33	(0.16;0.50)^*^	0.08
3.0–0.9% (RR 70%)	0.65	(0.42;0.89)^*^	0.65	(0.44;0.86)^*^	0.97
					
*Colonoscopy*					
*3.0–0.8% (RR 75%) (reference level)*					
3.0–0.5% (RR 85%)	0.16	(−0.03;0.35)	0.19	(0.02;0.36)^*^	0.79
3.0–0.1% (RR 95%)	0.40	(0.17;0.62)^*^	0.41	(0.20;0.61)^*^	0.95
					
*Screening interval*					
*FOBT*					
*4 monthly (reference level)*					
Annual	0.73	(0.52;0.93)^*^	0.64	(0.44;0.83)^*^	0.50
Triennial	0.96	(0.72;1.20)^*^	0.67	(0.46;0.89)^*^	0.07
					
*Sigmoidoscopy*					
*Annual (reference level)*					
5 yearly	0.92	(0.74;1.11)^*^	0.55	(0.39;0.72)^*^	<0.001
10 yearly	1.14	(0.91;1.37)^*^	0.56	(0.36;0.75)^*^	<0.001
					
*Colonoscopy*					
*Biennial (reference level)*					
5 yearly	0.71	(0.52;0.90)^*^	0.56	(0.39;0.73)^*^	0.22
10 yearly	0.72	(0.48;0.95)^*^	0.42	(0.21;0.63)^*^	0.06

Abbreviations: CI=confidence interval; CRC=colorectal cancer; FOBT=faecal occult blood test; RR=risk reduction.

^†^*P*-value describes the difference between screening-naive and previously screened subjects.

^*^P-value <0.05 compared with the reference level.

**Table A1:**
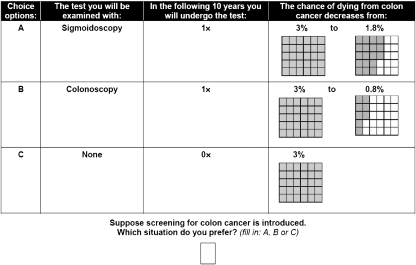
Choice set

**Table A2:** Background information on all screening test as applied to all subjects

	**Faecal occult blood test**	**Sigmoidoscopy**	**Colonoscopy**
Preparation	None.	- One or two enemas (bowel preparation). - No fasting.	- You have to drink 4 l of special cleansing solution the day before the procedure. - You have to fast for 12 h before the procedure. - You cannot work the afternoon before and the day of the procedure.
The procedure	**How do I carry out the test?** At home, you collect a small amount of faeces of 1–3 bowel movements using a test set (see picture). You can return the test by mail to the laboratory. **What does the test measure?** The test measures whether there are (in)visible traces of blood present in the stools. **What happens if the test results are abnormal?** You will be advised to undergo a colonoscopy.	**The procedure** The last 60 cm of the large bowel is examined by using a flexible tube with a small camera on the tip. This tube is inserted through the anus. During the procedure the large bowel will be filled with air to carefully examine the bowel. **What do I feel of the investigation?** Because of the air put into your bowel you may feel abdominal cramps. **What happens if abnormalities are found?** Precursors of colon carcinoma (polyps) are removed during the procedure (this is painless). You will be advised to undergo a colonoscopy to see whether there are other abnormalities in the remainder large bowel.	**The procedure** You will be given conscious sedation (‘short narcosis’). Therefore, you may fall into a light sleep. The entire large bowel (100-120 cm) is examined by using a flexible tube with a small camera on the tip. This tube is inserted through the anus. During the procedure the large bowel will be filled with air in order to carefully examine the bowel. **What do I feel of the investigation?** Due to the air and tube in your bowel you may feel abdominal pressure and cramps. **What happens if abnormalities are found?** Precursors of colon carcinoma (polyps) are removed during the procedure (this is painless).
After the procedure	- You can return to your daily activities immediately.	- You may eat and drink again immediately and go home.	- You may eat and drink again and go home after one hour. - You cannot drive a car, ride a motorcycle or bicycle.
Perceived burden	Low.	High.	High.
Results	- You will receive the result by mail within 2 weeks.	- Directly after the procedure. - When tissue has been removed, you will receive the pathology results by mail within 2 weeks.	- Directly after the procedure. - When tissue has been removed, you will receive the pathology results by mail within 2 weeks.
Test at home or in the hospital	At home.	Hospital.	Hospital.
Total duration of the procedure	30 min.	15 min.	1 h and 45 min.
Complications	Never.	In 1 in 10,000 individuals: severe blood loss or a perforation or a tear through the bowel wall.	In 1 in 1000 individuals: severe blood loss or a perforation or a tear through the bowel wall.

## Appendix

Table A1 and A2
